# External Beam Radiation in Differentiated Thyroid Carcinoma

**DOI:** 10.5041/RMMJ.10235

**Published:** 2016-01-28

**Authors:** Salem Billan, Tomer Charas

**Affiliations:** 1 Department of Oncology, Princess Margaret Hospital, Toronto, Canada and; 2 Division of Oncology, Rambam Health Care Campus, Haifa, Israel

**Keywords:** Carcinoma, external beam, radiotherapy, thyroid

## Abstract

The treatment of differentiated thyroid carcinoma (DTC) is surgery followed in some cases by adjuvant treatment, mostly with radioactive iodine (RAI). External beam radiotherapy (EBRT) is less common and not a well-established treatment modality in DTC. The risk of recurrence depends on three major prognostic factors: extra-thyroid extension, patient’s age, and tumor with reduced iodine uptake. Increased risk for recurrence is a major factor in the decision whether to treat the patient with EBRT. Data about the use of EBRT in DTC are limited to small retrospective studies. Most series have demonstrated an increase in loco-regional control. The risk/benefit from giving EBRT requires careful patient selection. Different scoring systems have been proposed by different investigators and centers. The authors encourage clinicians treating DTC to become familiarized with those scoring systems and to use them in the management of different cases. The irradiated volume should include areas of risk for microscopic disease. Determining those areas in each case can be difficult and requires detailed knowledge of the surgery and pathological results, and also understanding of the disease-spreading pattern. Treatment with EBRT in DTC can be beneficial, and data support the use of EBRT in high-risk patients. Randomized controlled trials are needed for better confirmation of the role of EBRT.

## INTRODUCTION

Thyroid cancer is one of the most common malignancies throughout the world, with incidence and prevalence on the rise in recent years in Israel and worldwide.^1^,^2^ Most thyroid cancers originate from glandular epithelial cells and are divided by their histological malignancy source into papillary (PTC), follicular (FTC), and anaplastic (ATC) thyroid carcinomas, with prevalences of 80%, 15%, and ∼2%, respectively.^3^

The treatment of differentiated thyroid carcinoma (DTC) is primary surgery (hemithyroidectomy or total thyroidectomy, with or without neck dissection), followed by adjuvant treatment given depending on the stage and risk profile of the tumor, most commonly radioactive iodine (RAI) in medium- and high-risk patients.^4^,^5^

Differentiated thyroid carcinoma is a radiosensitive cancer, proven by the effectivity of using radiation treatment in the form of RAI. Another form of radiation treatment, external beam radiotherapy (EBRT), a term used when radiation originates from an external source, is one of the pillars of oncological treatments for cancers; however, it is less commonly used in the treatment of DTC.

We present the relevant literature and indications for EBRT in the treatment of DTC. We discuss the prognostic factors for loco-regional relapse and the role of EBRT in reducing the risk of loco-regional recurrence in the presence of such prognostic factors.

## PROGNOSTIC FACTOR FOR LOCAL RECURRENCE

Cancer recurrence occurs in 10%–15% of patients with DTC and is most frequently confined to the neck.^6^ The risk of recurrence depends on three major prognostic factors: extra-thyroid extension, patient’s age, and tumor with reduced iodine uptake.^7^–^10^

### Extra-thyroid Extension

Extra-thyroid extension is a known risk factor for recurrence and death from DTC. In approximately 10% of DTC cases, the primary tumor extends through the thyroid gland capsule, leading to a poor prognosis, with death rates in the range of 5%– 35%.^11^ In a report by Loh et al., patients with extra-thyroid extension (stage T4) had more than a 3-fold risk of recurrence and death compared with those with large but thyroid-confined (stage T3) tumors.^12^ The effectiveness of adjuvant RAI treatment is reduced in the presence of extra-thyroid extension and extra-thyroidal disease.

### Patient’s Age

The risks of both recurrence and death increase also with increased patient’s age. This can partially be explained by the fact that tumors in older patients are less differentiated and are less likely to concentrate RAI.^13^,^14^

### Reduced Iodine Uptake

Reduced iodine uptake is also associated with a worse prognosis and increased risk of recurrence. Hürthle cell carcinomas, a known variant of PTC, have reduced RAI uptake compared to other PTC variants or follicular carcinomas. In a series of 101 patients with distant metastases, RAI uptake by pulmonary metastases was demonstrated in 64% of follicular carcinomas and 60% of papillary carcinomas, compared to only 36% of Hürthle cell carcinomas.^15^

## EBRT THERAPY

The use of external beam radiotherapy in the majority of solid cancer types increases local control, decreases the risk of recurrence, and even improves survival.^16^ The role of EBRT in DTC remains controversial due to the lack of randomized controlled trials and the reliance on single institution experiences and retrospective reviews, which include data that are usually biased for inconsistency in pathology assessments, surgical technique, and radiation therapy techniques, doses, and target definitions. We used the PubMed database for our primary search, which included EBRT and differentiated or papillary carcinoma search words. We employed the reference lists of the full-text articles that we retrieved electronically. Studies that reviewed the topic in terms of EBRT outcomes were included. Considering the level of evidence of EBRT efficacy in DTC and potential EBRT morbidity (including mucositis, pharyngitis, xerostomia, thick saliva, skin fibrosis, tracheal stenosis, and esophageal stricture), the decision of treatment for each case is challenging, which has brought many institutions and leading physicians to develop a “case-by-case” approach.

Treatment with EBRT has been known to control unresectable and gross residual thyroid cancer at least since the 1960s. Chow et al.^17^ reported a large retrospective cohort study and found a statistically significant improvement in loco-regional control in the subgroup of patients with macroscopic non-palpable residual disease treated with EBRT compared to those not irradiated (10-year loco-regional control rate 79.2% with EBRT versus 39% without EBRT, *P*<0.001). This study revealed a statistically significant increase in 10-year cause-specific survival (CSS) (74.1% with EBRT, 49.7% without EBRT, *P*=0.01) after treatment with EBRT. Similar long-term control of gross disease has been shown by others.^8^

Treatment with EBRT can also increase local control and local failure-free rates in microscopic disease. Several reports of retrospective reviews, in which local control was improved after EBRT in patients with high-risk microscopic disease compared with patients who did not receive EBRT, are listed in Table 1.

**Table 1.** Ten-year Loco-regional Control Rates in the High-risk Differentiated Thyroid Carcinoma Patients without Gross Residual Disease.

**First Author**	**Year of Publication**	**Surgery/RAI/EBRT (%)**	**Surgery/RAI (%)**
Tubiana et al.^18^	1985	86	79
Simpson et al.^19^	1990	86	82
Philips et al.^20^	1993	97	79
Farahati et al.^21^	1996	90	50
Tsang et al. ^22*^	1998	93	78
Ford et al.^23^	2003	82	37
Kim et al.^24^	2003	95	63.5
Brierley et al.^25†^	2005	86	65
Keum et al.^26^	2006	72	11
Meadows et al.^27^	2006	89	N/A
Terezakis et al.^28^	2009	75	N/A
Schwartz et al.^29^	2009	79	N/A

* Papillary only;

† Patients >60 y.

The most convincing report is from a study by Farahati et al.^21^ that reviewed 125 DTC patients with a high risk of local recurrence due to extra-thyroid extension and patient age over 40 years. All patients received the same treatment, including surgery followed by RAI therapy and thyroid-stimulating hormone suppression. The addition of EBRT improved the 5-year loco-regional control from 60% to 90%; EBRT was a predictive factor for improvement in time to distant failure (*P*<0.0003).

A study by Brierley et al.^30^ demonstrated improvement in loco-regional control and statistically significant better outcomes in patients over the age of 60 years receiving EBRT for thyroid carcinoma with microscopic residual disease, T4 disease, and no metastases. The 10-year loco-regional relapse-free rate was 86.4% with EBRT versus 65.7% without EBRT (*P*<0.01).

Other studies have reported significantly improved loco-regional control in similar subgroups with advanced local disease and/or postoperative residual disease.^21^,^22^ In contrast, two large retrospective reviews failed to show any difference in loco-regional control in patients who had received EBRT compared with those who had not.^22^,^31^

A recently published systematic review by Fussey et al.^32^ confirms the improvement in loco-regional control with EBRT in the older patient population with high-risk features.

## PATIENT SELECTION FOR EBRT

The potential benefit from the addition of EBRT needs to be balanced with the generally excellent outcomes with surgery and RAI, as well as the expected side effects of EBRT. Another key issue is whether improvement in local control only is sufficient to indicate irradiation, given the fact that local relapses may be salvaged with surgery and RAI.

To better define the subgroup of patients who would benefit the most from EBRT, different scoring systems were developed.^33^,^34^ Detailed in Table 2 is the scoring system proposed by a French group that is currently being tested in a prospective study. This scoring system takes into account classical prognostic factors and histology, gender, and recurrent disease. In cases with scores above 4, EBRT should be discussed, and, in scores above 6, EBRT is recommended.^35^

**Table 2.** Proposed Scoring System for Differentiated Thyroid Carcinoma.^23^

	**0 Point**	**1 Point**	**2 Points**	**4 Points**
Age (years)	<45	45–60	>60	
Gender	Female	Male		
Resectability	Resectable			Unresectable
Extensive extra-capsular nodal spread	No		Yes	
Extra-thyroidal disease		T3	T4	
R0–R1–R2	R0		R1	R2
Histological variants (tall-cell, Hürthle cell, columnar, diffuse sclerosing, solid variant)	No	Yes		
Recurrent disease (risk of recurrent nerve damage, etc.)	No		Yes	
Tumors with radioiodine fixation	Yes			No

Patient selection for EBRT can be challenging, and the authors’ opinion is that cases should be discussed in multidisciplinary tumor boards that include surgeons, endocrinologists, specialists from the radiation and medical oncology fields, diagnostic specialists (radiology and nuclear medicine), and pathologists. In our hospital, we recommend EBRT for patients older than 60 years of age with non-radioactive avid residual macroscopic disease that was considered not operable. Patients older than 60 years of age with microscopic residual disease are likely to develop an early recurrence that will most likely be non-operable and unlikely to respond to RAI.

## RADIATION VOLUME, DOSE, AND TECHNIQUE

There is no clear recommendation regarding radiation volume. At Rambam Health Care Campus, the irradiated volume depends on the disease spread pattern. In cases with locally advanced disease with extra-thyroid extension without lymph node involvement, the volume includes the thyroid bed and only the immediate adjacent lymph node areas (commonly levels 6 and 4). In cases with multiple lymph node involvement, the irradiated volume would include most of the lymph node levels, including levels 2, 3, 4, 5 bilaterally, level 6, and upper mediastinum, in addition to the tumor bed. This volume is adjusted as required depending on the preoperative imaging, the report of the surgeon at the time of resection, and the pathology report.

The EBRT dose is typically in the range of 60–66 Gy to areas of microscopic disease. When resection is incomplete, the dose to regions of known gross disease is increased to 70 Gy. Often, the energy used is 6 MV; however, when 3-dimensional planning is done, coverage of deeper structures can be achieved using 18 MV beam energy.

Achieving loco-regional control is paramount in patients treated with EBRT. Therefore, it is important to deliver a high dose of radiation, without causing injury to the surrounding normal tissues. This is can be achieved relatively easily using modern radiation techniques, such as intensity-modulated radiation therapy (IMRT). In a comparison study between IMRT and 3-dimensional conformal radiotherapy treatment plans, the Royal Marsden Hospital reported that the IMRT plan resulted in improved coverage of the planning volume and lower spinal cord dose.^36^

## CONCLUSIONS

Treatment with EBRT should be reserved for patients who are at high risk of loco-regional recurrence, and clinicians are urged to consider the potential benefits and possible toxicity. To date, there is sufficient evidence to support the use of EBRT in DTC. However, randomized control trials are warranted.

## Abbreviations:

ATC: anaplastic thyroid carcinoma
DTC: differentiated thyroid carcinoma
EBRT: external beam radiotherapy
FTC: follicular thyroid carcinoma
IMRT: intensity-modulated radiation therapy
PTC: papillary thyroid carcinoma
RAI: radioactive iodine.
